# Easy Access to Evans’ Oxazolidinones. Stereoselective Synthesis and Antibacterial Activity of a New 2-Oxazolidinone Derivative

**DOI:** 10.3390/molecules19067429

**Published:** 2014-06-06

**Authors:** Gaspar Diaz, Michelle A. A. de Freitas, Maria E. Ricci-Silva, Marisa A. N. Diaz

**Affiliations:** 1 Departamento de Química, Instituto de Ciências Exatas, Universidade Federal de Minas Gerais, Belo Horizonte–MG 31270-901, Brazil; E-Mail: adrianeamantea@gmail.com; 2 Departamento de Bioquímica e Biologia Molecular, Universidade Federal de Viçosa, Viçosa–MG 36570-000, Brazil; E-Mails: esthericci@hotmail.com (M.E.R-S.); marisanogueira@ufv.br (M.A.N.D.)

**Keywords:** Evans’ oxazolidinones, stereoselective synthesis, Morita-Baylis-Hillman adduct, *Staphylococcus aureus*, mastitis bovina

## Abstract

An interesting new approach was developed for the synthesis of Evans’ chiral auxiliaries with excellent yields. In turn, another new stereoselective and efficient strategy has also allowed for the preparation of a 2-oxazolidinone derivative in 34% overall yield from the Morita-Baylis-Hillman adduct. The antibacterial activity of this oxazolidinone was tested against *Staphylococcus aureus* strains isolated from animals with mastitis infections.

## 1. Introduction

### 1.1. Evans’ Oxazolidinones

**Figure 1:**
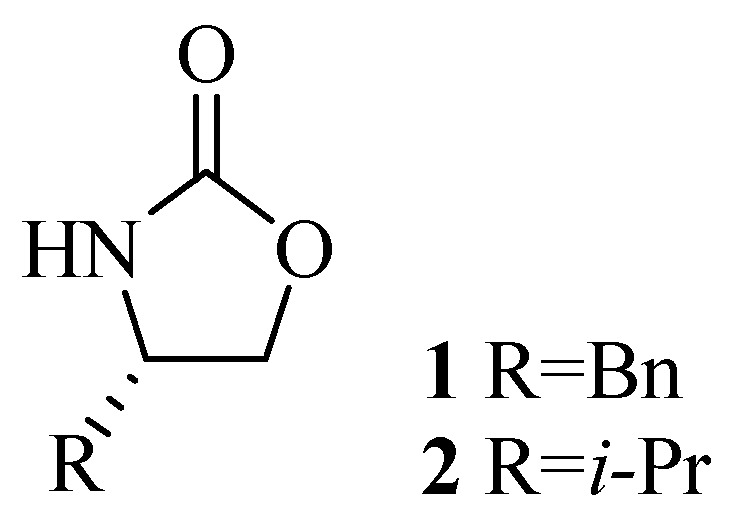
Structures of the Evans’ oxazolidinones **1** and **2**.

The use of enantiomerically pure oxazolidin-2-one derivatives as chiral auxiliaries in asymmetric aldol condensations was first reported by Evans *et al.* [1], in 1981 (Figure 1), and the enormous utility of these and related oxazolidinones has been amply demonstrated [2,3,4,5].

Many sources for oxazolidin-2-one structures have been documented; however, preparative access to chiral auxiliaries has proven to be only moderately efficient due to the β-aminoalcohol cyclization step using phosgene, urea, or diethyl carbonate, which gives moderate yields [6]. Of the procedures used for amino-alcohols cyclization, those involving phosgene proved to be most convenient and produced the best yields. However, phosgene has proven to be a dangerous gas for humans, therefore, other alternative methods are needed to properly prepare these oxazolidinones.

Therefore, this study describes a novel, short, and direct alternative strategy, for the preparation of the oxazolidinones **1** and **2**, in nearly quantitative yields and without the purification of intermediates.

### 1.2. Oxazolidinone Derivatives

**Figure 2:**
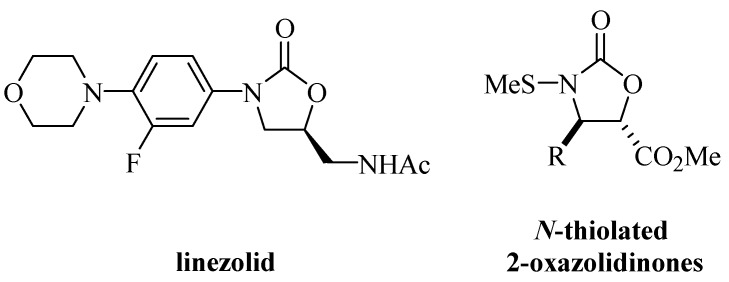
Structures of the linezolid and *N*-thiolated 2-oxazolidinones.

Several compounds containing 4-substituted and 4,5-disubstituted oxazolidin-2-one rings have shown promising pharmacological activities. For instance, in the 1980s, DuPont researchers found that certain compounds containing 2-oxazolidinone rings showed potent antibacterial activities through a unique mechanism of action [7]. Linezolid (Figure 2) was the first 2-oxazolidinone derivative with antibiotic action to be introduced into clinical trials by Pharmacia [8] and was approved in 2000 by the Food and Drug Administration for use in the treatment of infections caused by Gram-positive bacteria.

Turos *et al.* [9] recently reported the discovery of compounds with *N*-thiolated 2-oxazolidinone rings (Figure 2) as a new family of antibacterial agents, since many of these *N*-thiolated derivatives showed a potent antibacterial activity against methicillin-resistant *Staphylococcus aureus* (MRSA) [10].

In conjunction with the first portion of this work, and as part in another ongoing research work aimed at the synthesis of natural products with potential antibacterial activities, the present study also proposes a novel approach for the stereoselective synthesis of a new 2-oxazolidinone derivative **5**. This was possible following the same strategy of *N*,*O*-heterocyclization of the oxazolidinones **1** and **2**, respectively, from diol **12**. Thus, as a supplement to the work previously published by Coelho *et al.* [11], the synthesis of this 2-oxazolidinone provided the opportunity to explore the conversion of a Morita-Baylis-Hillman (MBH) adduct as a key intermediate into 4,5-disubstituted 2-oxazolidinone **5**. To the best of our knowledge, this approach is the first that examines the major MBH stereoisomer, which in turn produced the acetonide derivative **14** as an advanced intermediate used to synthesize 2-oxazolidinone products.

## 2. Results and Discussion

### 2.1. Preparation of Evans’ Oxazolidinones **1** and **2**

**Scheme 1:**
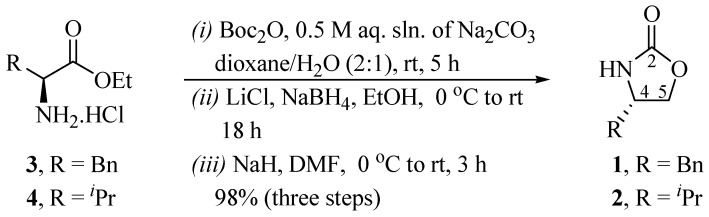
Preparation of Evans’ oxazolidinones **1** and **2**.

The commercially available L-phenylalanine ethyl ester hydrochloride **3** was converted to the corresponding *N*-Boc ethyl ester in the presence of di-*tert*-butyl dicarbonate ((Boc)_2_O), followed by the reduction of the ester function using LiBH_4_. The cyclization was performed by treating the resulting alcohol with NaH, producing the corresponding oxazolidin-2-one derivative **1** with a yield of 98% (for three steps), [α]*_D_*^25^ −62.5 (*c* 1.0, CHCl_3_) (Scheme 1).

In a similar fashion to **1**, the commercially available L-valine ethyl ester hydrochloride **4** was transformed into oxazolidinone **2** (98%, three steps), [α]*_D_*^25^ −15.3 (*c* 7.0, CHCl_3_) (Scheme 1); the characterization data of both **1** and **2** are in good agreement with reported values [1].

### 2.2. Preparation of 2-Oxazolidinone Derivative **5**

**Scheme 2:**
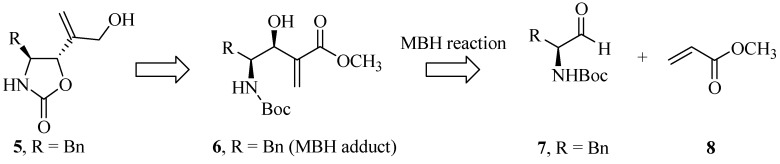
Retrosynthetic analysis of 2-oxazolidinone derivative **5**.

Initially, the stereoselective synthesis of 2-oxazolidinone derivative **5** was proposed according to the retrosynthetic analysis shown in Scheme 2. The Morita-Baylis-Hillman adduct **6** was obtained according to the procedure described by Coelho *et al.* [11]. *N*-Boc protection of L-phenylalanine ethyl ester hydrochloride **3** using di-*tert*-butyl dicarbonate and reduction of the ester function of crude product by treating with DIBAL-H at −78 °C produced aldehyde **7** (note: the crude aldehyde was used immediately after preparation to avoid or minimize racemization). The L-phenylalaninal **7** was treated with DABCO and methyl acrylate and was kept in an ultrasonic bath for 36 h to produce the diastereomeric mixture (3:7; *anti:syn*) according to chiral gas chromatography (see Supplementary Material for details). The crude mixture was purified by column chromatography to produce the major Morita-Baylis-Hillman adduct **6** (56%, 3 steps) in 99% ee as determined by chiral GC.

**Scheme 3:**
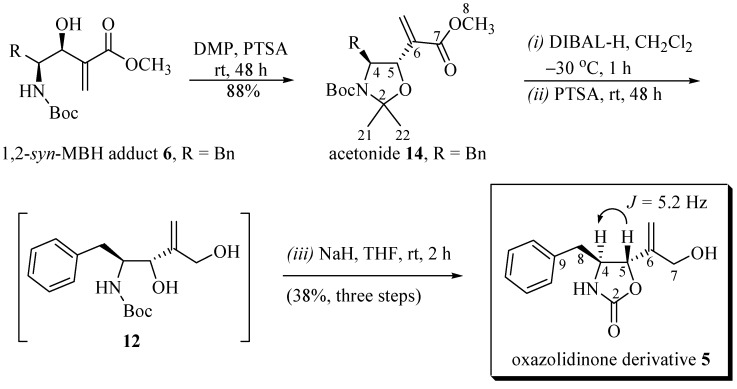
Synthesis of 2-oxazolidinone derivative **5** from 1,2-*syn-*MBH adduct **6**.

At first glance, the *N,O*-heterocyclization from the MBH adduct **6** appeared to be quite simple. However, the preliminary results contradicted this prediction. Any attempt, including *inter alia* direct*N,O*-heterocyclization from this adduct with the intact ester function and free hydroxyl led to a complex mixture of products, as witnessed when **6** was treated with NaH or phosgene, for example. Due to the unsatisfactory results obtained from the initial proposal to attempt the direct *N,O-*heterocyclization from MBH adduct **6**, a new route was proposed, this time in four steps from the adduct **6**, as shown in Scheme 3.

The step performed to protect the Morita-Baylis-Hillman adduct **6** had to be radically modified for the *N,O*-protected acetonide derivative (Scheme 3). For this purpose, this adduct was treated with 2,2-dimethoxypropane and catalytic amounts of *p-*toluenesulfonic acid, producing the acetonide derivative **14** in 88% yield. The one pot sequence, starting from the reduction of the ester function of acetonide **14** using DIBAL at −30 °C, a chemoselective deprotection using *p*-toluenesulfonic acid, and cyclization of the resultant diol **12** in the presence of NaH produced the corresponding oxazolidinone derivative **5**with a yield of 38% (three steps). The relative stereochemistry of *trans-*oxazolidinone derivative **5** can readily be assigned by comparing it to the ring-proton coupling constant (*J*_H5-H4_) [12,13,14]. The observed coupling constant of *J*_H5-H4_ = 5.2 Hz indicates that the protons are on opposite faces of the heterocyclic ring; therefore, the *trans-*oxazolidinone **5** is derived from the 1,2-*syn* MBH adduct **6** (Scheme 3).

### 2.3. Antibacterial Activity

Compounds **5** and **14** were assayed against *Staphylococcus aureus* strains. Ciclopirox olamine was used as positive control since this compound possesses a broad-spectrum of antibacterial and anti-inflammatory activities [15,16] and it is one of the most used drugs to treat bovine mastitis in some farms in Brazil. According to Table 1, oxazolidinone derivative **5** has shown good antimicrobial activity against the five *S. aureus* strains isolated from animals with bovine mastitis infection. However, the results of bioassays performed on acetonide **14**showed little or no activity. These results are in agreement with findings from DuPont researchers [7,8] and Turos *et al.* [9] who affirm that some compounds containing oxazolidinone rings present a potent antimicrobial activity against methicillin-resistant *S. aureus* (MRSA) and other Gram-positive bacteria. It is believed that, in addition to the substituents on carbons 4 and 5, respectively, this activity is likely related to the presence of a more rigid and almost planar ring. This ring rigidity is more probable in the presence of a carbonyl group at 2-oxazolidinone **5**, when compared with the ring of the acetonide derivative **14** (Scheme 3) since this most likely provides a more flexible conformation bias.

**Table 1:** Antibacterial activity of compounds **5** and **14** (inhibition zones in mm ± SD).

S. aureus	Compound 5	Compound 14	Ciclopirox Olamine	DMSO
680	19.0 ± 0.58	2.0 ± 0.48	17.0 ± 0.58	0.00
2221	18.0 ± 0.60	NA	18.0 ± 0.60	0.00
4006	21.0 ± 0.33	2.0 ± 0.58	17.0 ± 0.58	0.00
4075	15.0 ± 0.67	NA	17.0 ± 0.58	0.00
4127	16.0 ± 0.58	2.0 ± 0.50	17.0 ± 0.50	0.00

NA = not active.

Table 2 shows the results of the minimum inhibitory concentration (MIC) for compound **5** against *S. aureus* (4006 strain) which presented the best value in the antibacterial assay. The MIC value was lower than the standard antibiotic value, demonstrating that this compound, containing a 2-oxazolidinone ring, belongs to a new family of compounds with potential antimicrobial activity.

**Table 2:** MIC value obtained from compound **5** against *Staphylococcus aureus* (4006 strain).

MIC (μg mL^−1^)	
Compound 5	0.03
Ciclopirox olamine	0.05

## 3. Experimental

### 3.1. General Procedures

All reactions were carried out under N_2_ in flame-dried glassware with magnetic stirring. CH_2_Cl_2_, Et_3_N, and DMF were distilled from CaH_2_. THF was distilled from sodium/benzophenone ketyl. Chromatographic purification was carried out on silica gel (mesh particle size 70–230) and thin layer chromatography (TLC) was performed on silica GF_254_ Merck. TLC visualization was accomplished with UV light and by spraying with 7% ethanolic phosphomolybdic acid and heating. ^1^H- and ^13^C-NMR (nuclear magnetic resonance) spectra were recorded on a Varian Gemini BB at 300 MHz and 75.4 MHz, respectively. FTIR spectra were determined using KBr disks on a FTIR Perkin Elmer Spectrum 200 spectrometer. Uncorrected melting points were determined using METTLER equipment, model FP82. GC analyses were recorded on HP6890 equipment with a flame ionization detector, using an HP-5 capillary (cross-linked 5% phenylmethylsiloxane, 30 m, 0.25 mm × 25 µm) column (Agilent Technology, Santa Clara, CA, USA). Mass spectra were recorded on a Prominence HPLC (Shimadzu, Kyoto, Japan) coupled to the MicroQTOF II mass spectrometer (Bruker Daltonics, Fremont, CA, USA). Optical rotation was measured through Perkin-Elmer equipment using a Na lamp at 585 nm. Diastereoselectivity of MBH adduct **6** was determined from GC analysis. The Morita-Baylis-Hillman reaction was sonicated in a UNIQUE model GA 10.25 (1200 W, 25 kHz) ultrasonic bath.

### 3.2. Synthesis

#### 3.2.1. General Procedure for the Synthesis of Evans’ Oxazolidinones **1** and **2**

To the respective L-amino ethyl ester hydrochloride (1 mmol) dissolved in dioxane-distilled water (2:1, 3.9 mL), 0.5 mol L^−1^ of an aqueous solution of Na_2_CO_3_ (4 mL) was added at room temperature. The resulting solution was cooled to 0 °C, at which time the di-*tert*-butyldicarbonate (192 mg, 1.1 mmol) was added and the ice-bath was removed. This mixture was stirred for 3 h at room temperature and extracted with EtOAc (3 × 10 mL). The combined organic layers were dried with MgSO_4_, filtered, and concentrated to give a slightly yellow solid that was dissolved in EtOH (4 mL). NaBH_4_ (113.5 mg, 3.0 mmol) and LiCl (127.2 mg, 3.0 mmol) were added to this flask at 0 °C, at which time the ice-bath was removed. This mixture was stirred for 18 h at room temperature and quenched by the addition of a saturated aqueous solution of citric acid (5 mL) followed by Et_2_O (10 mL). The layers were separated, and the aqueous layer was additionally extracted with Et_2_O (2 × 10 mL). The combined organic layers were treated with a saturated aqueous solution of NaHCO_3_ (5 mL), washed with brine (5 mL), dried with MgSO_4_. The solvent was concentrated to produce a slightly yellow solid, which was dissolved in DMF (3 mL), stirred, and cooled at 0 °C. Next, NaH (72 mg, 3.0 mmol) was added, and the resulting mixture was warmed gradually to room temperature, stirred for 3 h, and subsequently quenched by adding a 5% aqueous solution of hydrochloric acid (5 mL) followed by Et_2_O (10 mL). The layers were separated, and the aqueous layer was extracted using Et_2_O (2 × 10 mL). The combined organic layers were treated with a saturated aqueous solution of NaHCO_3_ (5 mL), washed with brine (5 mL), dried with MgSO_4_, and concentrated. The residues were separately purified by column chromatography on silica gel using 60% EtOAc/hexanes as the eluent to produce compounds **1** and **2**, respectively, with an average yield of 98% after the three steps.

*(S)-(−)-4-Benzyl-2-oxazolidinone* (**1**). [α]*_D_*^25^ −62.5 (*c* 1.0, CHCl_3_); mp 89–90 °C; IR (KBr) ν_max_/cm^−1^ 3280, 3023, 2853, 1960, 1752, 1548, 1475, 1454, 1403, 1021, 943; ^1^H-NMR (CDCl_3_) *δ*7.24–7,37 (m, 3H, H9/H9'/H10), 7.17–7,19 (m, 2H, H8/H8'), 5.78 (brs, 1H, NH), 4.44 (dd, *J* = 12.0, 8.1 Hz, 1H, H5), 4.04–4.14 (m, 2H, H4/H5), 2.87 (d, *J* = 6.3 Hz, 2H, H6); ^13^C-NMR (CDCl_3_) *δ* 159.6 (C2), 136.2 (C7), 129.2 (C8/C8'/C9/C9'), 127.5 (C10), 69.8 (C5), 54.0 (C4), 41.6 (C6).

*(S)-(−)-4-Isopropyl-2-oxazolidinone* (**2**). [α]*_D_*^25^ −15.3 (*c* 7.0, CHCl_3_); mp 72–73 °C; IR (neat) ν_max_/cm^−1^ 3259, 2960, 2914, 1720, 1471, 1404, 1327, 1242, 1089, 1007, 933, 706; ^1^H-NMR (CDCl_3_) *−*7.39 (brs, 1H, NH), 4.44 (dd, *J* = 8.6, 6.4 Hz, 1H, H5), 4.10 (dd, *J* = 8.6, 6.4 Hz, 1H, H5), 3.57–3.68 (m, 1H, H4), 1.64–1.81 (m, 1H, H6), 0.96 (d, *J* = 6.6 Hz, 3H, H7'), 0.90 (d, *J* = 6.6 Hz, 3H, H7); ^13^C-NMR (CDCl_3_) *δ* 160.6 (C2), 68.4 (C5), 58.2 (C4), 32.5 (C6), 17.7 (C7'), 17.4 (C7).

#### 3.2.2. (−)-(3S,4S)-4-Tert-butoxycarbonylamino-3-hydroxy-2-methylene-5-phenylpentanoic Acid Methyl Ester (**6**) [11]

To a solution of L-phenylalanine ethyl ester hydrochloride (**3**, 400 mg, 1.74 mmol) in MeOH (8.8 mL) at room temperature, di-*tert*-butyl dicarbonate (456 mg, 2.08 mmol), and NaHCO_3_ (432 mg, 5.2 mmol) were added. The resultant mixture was kept in an ultrasonic bath for 4 h at room temperature. The solvent was concentrated under reduce pressure. The residue was redissolved in Et_2_O (40 mL) and treated with a saturated aqueous solution of NaHCO_3_ (20 mL). The layers were then separated, and the aqueous layer was extracted with Et_2_O (3 × 20 mL). The combined organic layers were washed with brine (20 mL) and dried over MgSO_4_. After removal of the solvent, the crude *N*-Boc amino ester was obtained in a quantitative yield. To the crude carbamate dissolved in anhydrous toluene (5 mL), kept under an argon atmosphere, and cooled at −78 °C, DIBAL-H (1.3 mL, 1.9 mmol, 1.5 mol L^−1^ in toluene) was slowly added. The reaction mixture was additional stirred over 30 min at −78 °C and quenched by adding a methanol (0.2 mL) and Rochelle solution (2 mL) at same temperature. The heterogeneous mixture was then stirred vigorously for 3 h at room temperature. The reaction mixture was filtered over Celite, washed with EtOAc (3 × 20 mL), dried over MgSO_4_, and concentrated under reduced pressure to afford the crude L-phenylalaninal (90% yield; the crude aldehyde was used immediately after preparation to avoid or minimize racemization). The L-phenylalaninal was treated with DABCO (100 mg, 0.89 mmol) and methyl acrylate (2 mL, 23 mmol) and was kept in an ultrasonic bath for 36 h at room temperature. After that, the reaction mixture was diluted in dichloromethane (20 mL). The mixture was then washed with a saturated solution of NaHCO_3_, a solution of 10% HCl, brine, and finally dried over anhydrous sodium sulfate. After solvent evaporation under reduced pressure, the diastereomeric mixture (3:7; *anti:syn*) according chiral gas chromatography, was purified by column chromatography on silica gel using 20% EtOAc/hexanes as an elutant to produce 326 mg (56%, 3 steps) of the major Morita-Baylis-Hillman adduct (**6**) as a slight yellow oil in 99% ee as determined by chiral GC. [α]*_D_*^25^ −1.80 (*c* 1.0, CHCl_3_); IV (neat) ν_max_/cm^−1^ 3404, 2979, 2930, 1714, 1694, 1509, 1440, 1172; ^1^H-NMR (CDCl_3_) *δ* 7.31–7.16 (m, 5H, Ph-H), 6.31 (brs, 1H, CH_2_), 5.92 (brs, 1H, CH_2_), 4.87 (sh, 1H, NH), 4.51 (brs, 1H, OCH), 4.03 (brq, 1H, *J*= 7.0 Hz, NCH), 3.71 (s, 3H, OCH_3_), 3.08 (sh, 1H, OH), 3.03–2.90 (m, 2H, Bn-H), 1.36 (brs, 9H, C(CH_3_)_3_); ^13^C-NMR (CDCl_3_) *δ* 166.3 (C_0_), 156.1 (C_0_), 140.3 (C_0_), 138.2 (C_0_), 129.1 (CH), 128.2 (CH), 126.2 (CH), 125.7 (CH_2_), 79.4 (C_0_), 70.7 (CH), 54.8 (CH), 51.9 (CH_3_), 38.1 (CH_2_), 28.3 (CH_3_); HRMS (ESI^+^) *m/z*, calcd. for C_18_H_25_NO_5_ [M+1]^+^: 336.1733, found: 336.1793.

#### 3.2.3. (+)-(4S,5S)-4-Benzyl-5-(1-Methoxycarbonylvinyl)-2,2-dimethyloxazolidine-3-carboxylic Acid Tert-Butyl Ester (**14**)

To a solution of the Morita-Baylis-Hillman adduct **6** (335.4 mg, 1 mmol) in 2,2-dimethoxypropane (3.0 mL, 24.4 mmol), camphorsulfonic acid (46.5 mg, 0.20 mmol) was added and the mixture stirred for 48 h at room temperature. The reaction was quenched by adding distilled water (10 mL) followed by Et_2_O (10 mL). Subsequently, both layers were separated. The organic layer was washed with a saturated aqueous solution of NaHCO_3_ (5 mL) and brine (5 mL). The organic layer was dried with MgSO_4_, filtered, and concentrated. Purification by flash chromatography on silica gel using 3% EtOAc/hexanes as the eluent produced compound **14** in 80% yield. [α]*_D_*^25^ +0.03 (*c* 10.0, CHCl_3_); IR (neat) ν_max_/cm^−1^ 3063, 2985, 2951, 2936, 1696, 1633, 1452, 1437, 1376, 1363, 1279, 1252, 1165, 1129, 1067, 851, 700; ^1^H-NMR (CDCl_3_) *δ* 7.10–7.28 (m, 5H, H11-15), 6.25 (brs, 1H, H6_a_'), 5.89 (brs, 1H, H6_b_'), 4.84 (brs, 1H, H5), 4.16 (brs, 1H, H4), 3,63 (brs, 3H, H8), 3.14 (brs, 2H, H9), 1.52 (s, 9H, H18-20), 1.50 (s, 3H, H21), 1.45 (s, 3H, H22); ^13^C-NMR (CDCl_3_) *δ* 166.1 (C7), 151.5 (C16), 138.0 (C6), 135.5 (C10), 130.0 (C12), 127.9 (C11), 126.6 (C6'), 125.7 (C13), 92.7 (C2), 80.1 (C17), 74.5 (C5), 60.2 (C4), 51.7 (C8), 36.9 (C9), 28.4 (C18-20), 27.2 (C21), 23.8 (C22); HRMS (ESI^+^) *m/z* calcd for C_21_H_29_NO_5_ [M+1]^+^ 376.2046, found 376.2120.

#### 3.2.4. (−)-(3S,4S)-4-Benzyl-5-(1-Hydroxymethylvinyl)-2-oxazolidinone (**5**)

To a stirred solution under argon of the corresponding acetonide derivative **14** (120 mg, 0.32 mmol) in 2 mL of dichloromethane at −30 °C, 1.2 mol L^−1^ in toluene of DIBAL-H (1 mL, 0.83 mmol) was slowly added. After 90 min of stirring, the solution was warmed to 0 °C, stirred for 30 min, and quenched by adding a methanol (0.15 mL) and Rochelle solution (1 mL). The heterogeneous mixture was then stirred vigorously for 3 h. Following the addition of water (5 mL) and Et_2_O (5 mL), the layers were separated. The aqueous layer was extracted with Et_2_O (3 × 5 mL). The combined organic extracts were dried over MgSO_4_ and evaporated under reduced pressure to give an oily residue. This residue was dissolved in methanol (3 mL). Next, *p-*toluenesulfonic acid (17.2 mg, 0.1 mmol) was added and stirred for 14 h at room temperature. The reaction mixture was dissolved in EtOAc (5 mL) and treated with a saturated aqueous solution of NaHCO_3_ (3 mL). The layers were then separated, and the aqueous layer was extracted with EtOAc (3 × 5 mL). The combined organic layers were washed with brine (5 mL) and dried with anhydrous Na_2_SO_4_. The solvent was concentrated to give a slightly yellow residual product that was dissolved in anhydrous THF (2 mL) and, stirred at 0 °C under argon. A suspension of NaH (24 mg, 1.0 mmol) in anhydrous THF (2 mL) was added in a dropwise manner to this mixture and stirred at 0 °C for 2 h. The reaction was quenched with H_2_O (3 mL), and the layers were separated. The aqueous layer was extracted with EtOAc (3 × 5 mL). The combined organic layers were washed with brine, dried with anhydrous Na_2_SO_4_, and concentrated. The residue was purified by column chromatography using EtOAc/hexanes 50% as an eluent to produce 28.4 mg (38% yield, 3 steps) of oxazolidinone derivative **5** as a slightly yellow oil in 99% ee as determined by chiral GC. [α]*_D_*^25^ −0.04 (*c* 9.0, CHCl_3_); IR (neat) ν_max_/cm^−1^ 3283, 3029, 2922, 2855, 1734, 1390, 1231, 1019, 918, 733; ^1^H-NMR (CDCl_3_) *δ* 7.18–7.34 (m, 5H, H10-14), 5.65 (s, 1H, NH), 5.24 (s, 1H, H6_a_'), 5.13 (s, 1H, H6_b_'), 4.82 (d, *J* = 5.2 Hz, 1H, H5), 4.14 (s, 2H, H7), 3.97 (brd, *J* = 6.4 Hz, 1H, H4), 2.96 (dd, *J* = 13.6, 4.8 Hz, 1H, H8_a_), 2.84 (dd, *J* = 13.6, 4.8 Hz, 1H, H8_b_), 2.13 (brs, 1H, OH); ^13^C-NMR (CDCl_3_) *δ* 158.9 (C2), 144.6 (C6), 136.0 (C9), 129.3 (C10), 128.9 (C11), 127.2 (C12), 114.3 (C6'), 82.2 (C5), 62.2 (C7), 58.7 (C4), 41.5 (C8); HRMS (ESI^+^) *m/z* calcd. for C_13_H_15_NO_3_ [M+H]^+^ 234.1052, found 234.1069.

### 3.3. Antibacterial Assays

#### 3.3.1. Bacterial Strains and Culture Media

The bacterial strains used in this study, which were isolated from animals with mastitis infections, were kindly provided by Embrapa Dairy Cattle/Laboratory of Milk Microbiology (Juiz de Fora, Minas Gerais, Brazil). Five *S. aureus* strains (680, 2221, 4006, 4075, and 4127) were used to determine the antimicrobial activity of compounds **5** and **14**. Bacteria were routinely cultured on brain heart infusion (BHI) agar at 37 °C for 16 h prior to the experiments. The cell concentration was adjusted to 10^6^ CFU mL^−1^ with an optical density set at 600 nm. Stock cultures were maintained in BHI agar containing 25% glycerol at −80 °C.

#### 3.3.2. Antibacterial Screening Assay

Hole-plate diffusion assay was initially performed to test the antibacterial activity of compounds. To accomplish this, the bacteria were cultivated overnight, and a suspension containing 10^6^ CFU mL^−1^was spread on plates containing Müeller-Hinton agar (Himedia^®^, Mumbai, India). Holes of approximately 5 × 3 mm were made in the agar and filled with 30 μL of the stock with 10 µg mL^−1^ for compounds **5** and **14**. After incubation at 37 °C for 24 h, inhibition zones were measured in millimeters and compared to the controls. The antibiotic ciclopirox olamine (Uci-Farma^®^, São Bernardo do Campo, SP, Brazil) was used as the positive control due to its antibacterial properties [15,16]. Dimethylsulfoxide (DMSO) was used as a negative control. Tests were performed twice in triplicate. The minimum inhibitory concentration (MIC) of compound **5** was determined by applying a broth microdilution method followed by incubation at 37 °C for 24 h and by observing media turbidity [17]. Tests were performed twice in triplicate.

## 4. Conclusions

The Evans’ chiral auxiliaries **1** and **2** were prepared in high yields (98%, three steps) through a simple and straightforward alternative strategy. It is worth mentioning that either of the chiral auxiliaries can be obtained in gram scale. By contrast, a novel stereoselective synthetic approach allowed for the preparation of oxazolidin-2-one derivative **5** with an overall yield of 34% in four steps, starting from the Morita-Baylis-Hillman adduct **6**.

The bioassay of oxazolidinone **5** against *Staphylococcus aureus* isolated from animals with bovine mastitis infections showed a potent antibacterial activity, with an MIC value of 0.03 μg mL^−1^. Further studies on the synthesis of other oxazolidinone derivatives beginning with different amino acids are currently in progress.

## Supplementary Materials

Supplementary materials can be accessed at: http://www.mdpi.com/1420-3049/19/6/7429/s1.
