# Supplementary material for: Easy Access to Evans’ Oxazolidinones. Stereoselective Synthesis and Antibacterial Activity of a New 2-Oxazolidinone Derivative
Source: Molecules. 2014 Jun 6;19(6):7429–39. doi: 10.3390/molecules19067429 (PMC6270906; doi:10.3390/molecules19067429)
Supplement: Supplementary file 1 [file molecules-19-07429-s001.pdf]

Supporting Information

Table of Contents

Copies of IV, mass, ^{1}H and ^{13}C-NMR spectra for compounds S1–S14

Copies of HPLC chromatograms for compounds **6** and **5** S15

Figure S1. ^{1}H-NMR spectrum (300 MHz, $CDCl_3$) of Evans' oxazolidinone **1**.

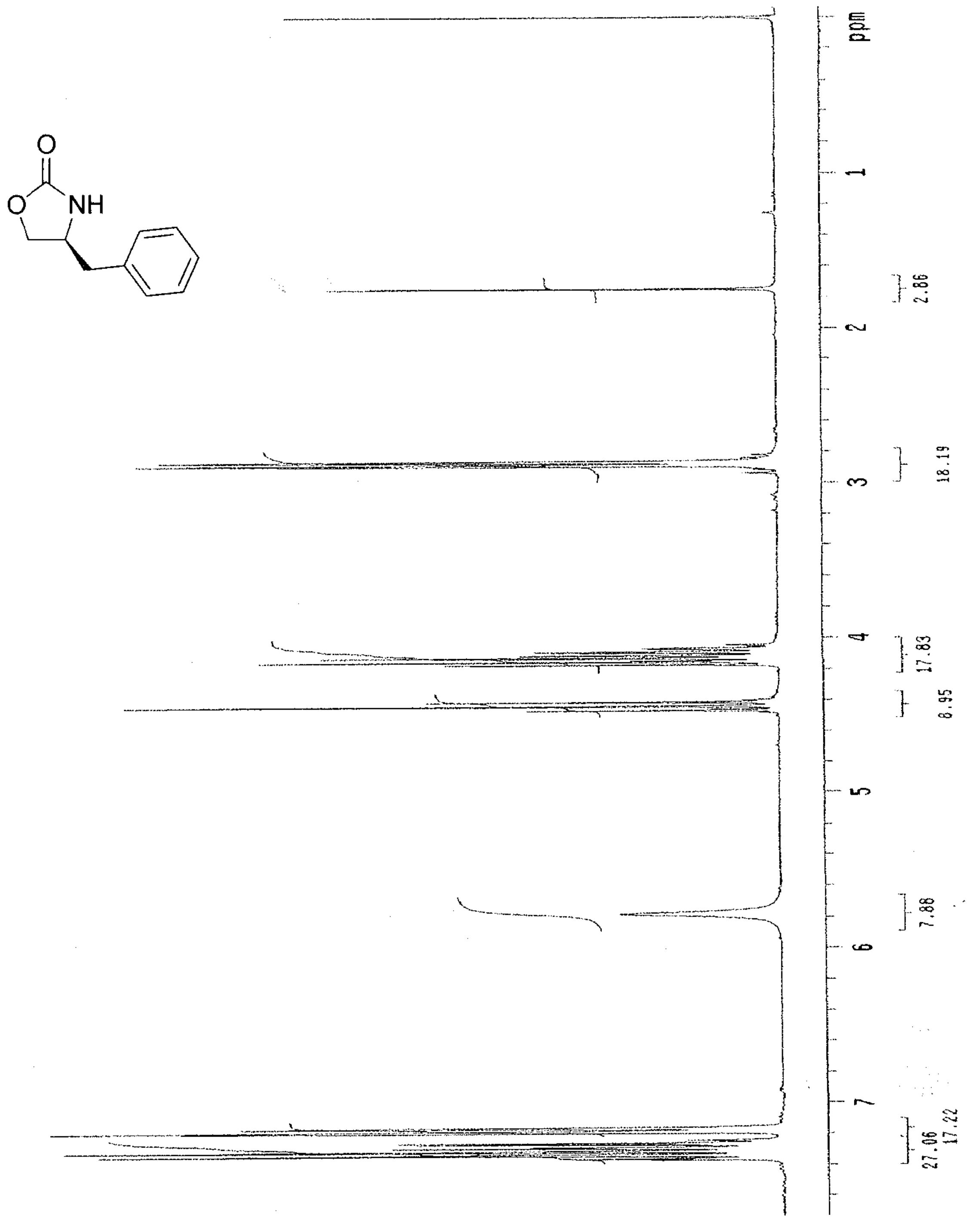

Figure S2. ^{13}C-NMR spectrum (75.4 MHz, $CDCl_3$) of Evans' oxazolidinone **1**.

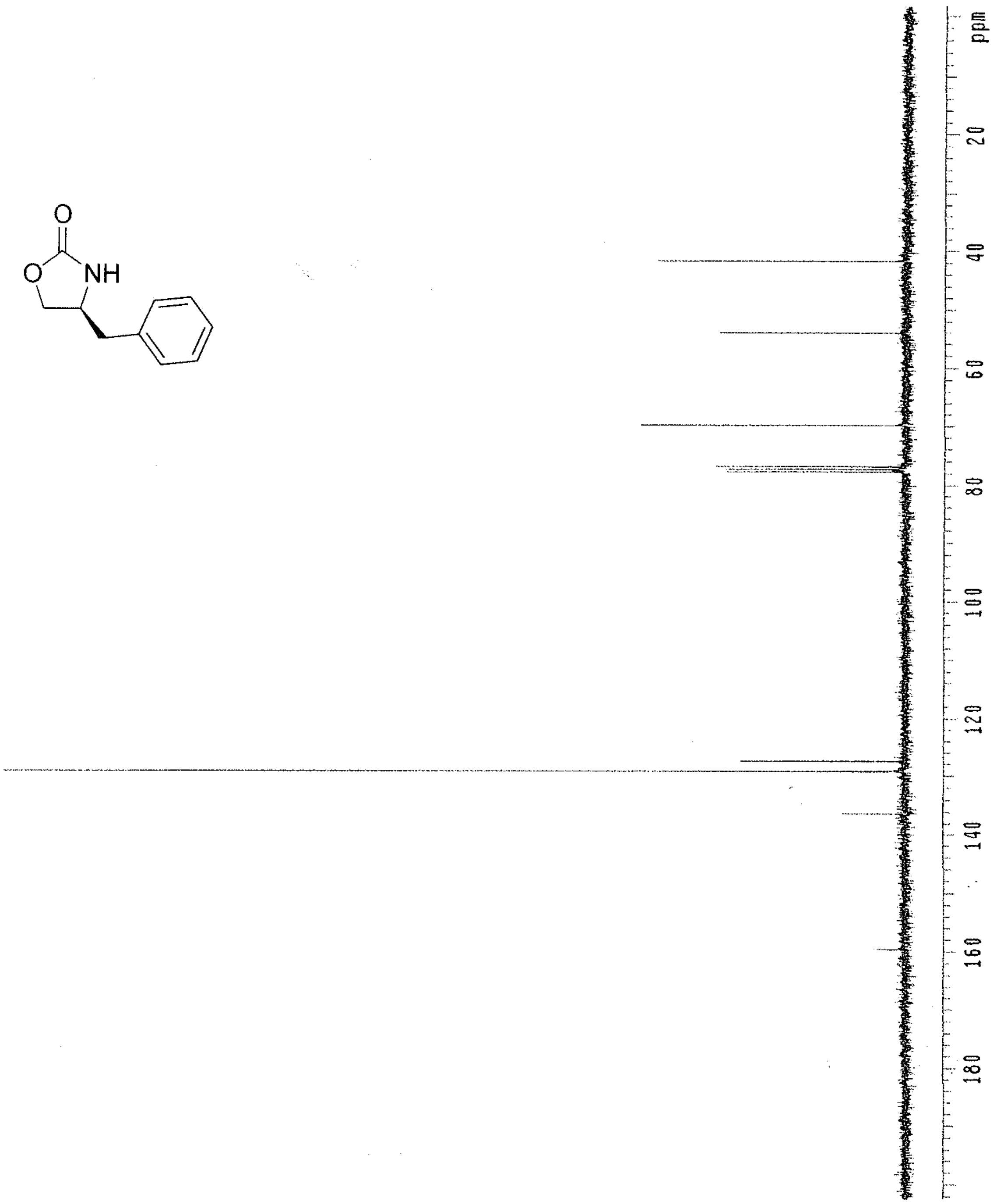

Figure S3. IV (KBr) spectrum of Evans' oxazolidinone **1**.

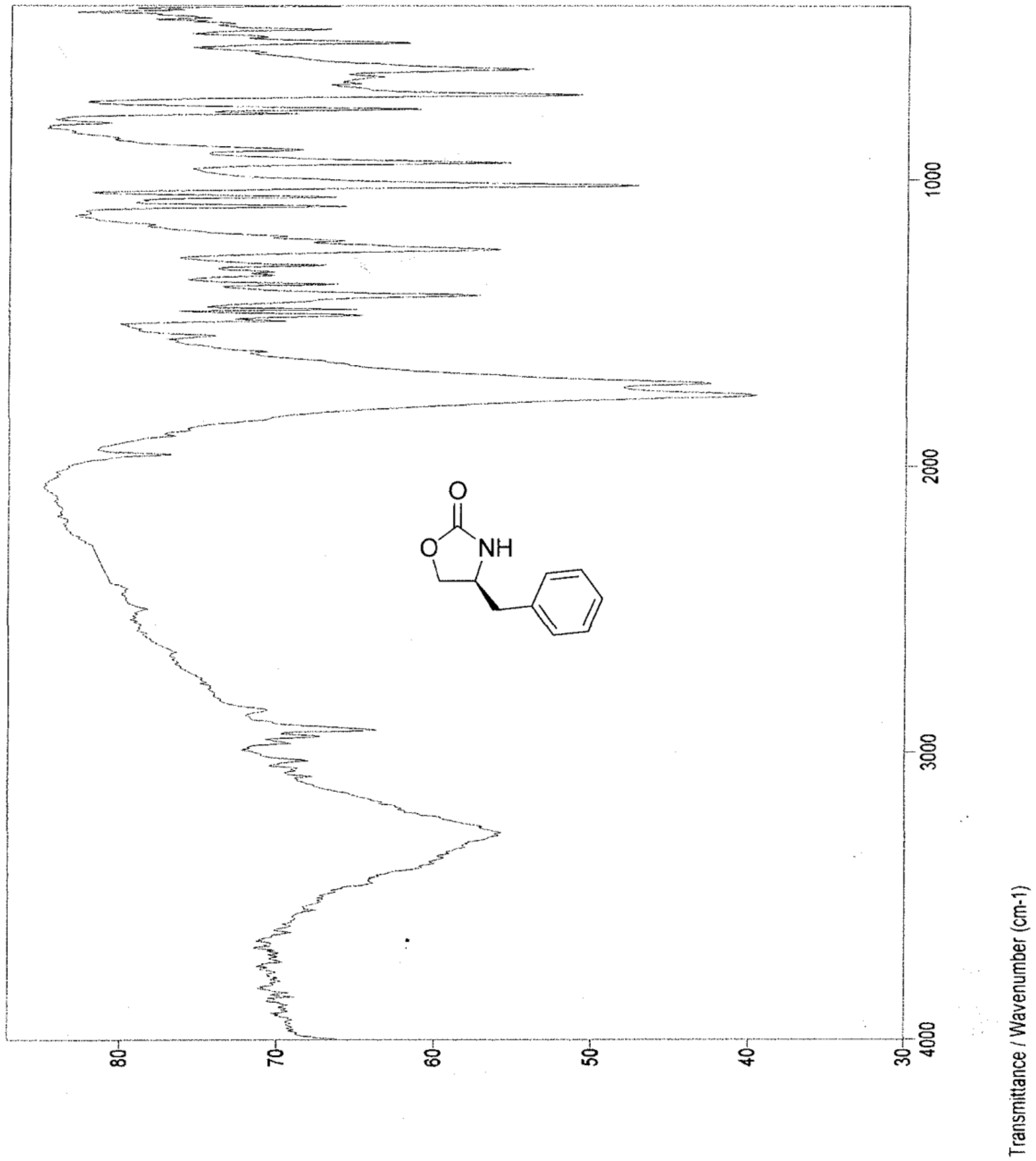

Figure S4. ^{1}H-NMR spectrum (300 MHz, $CDCl_3$) of Evans' oxazolidinone **2**.

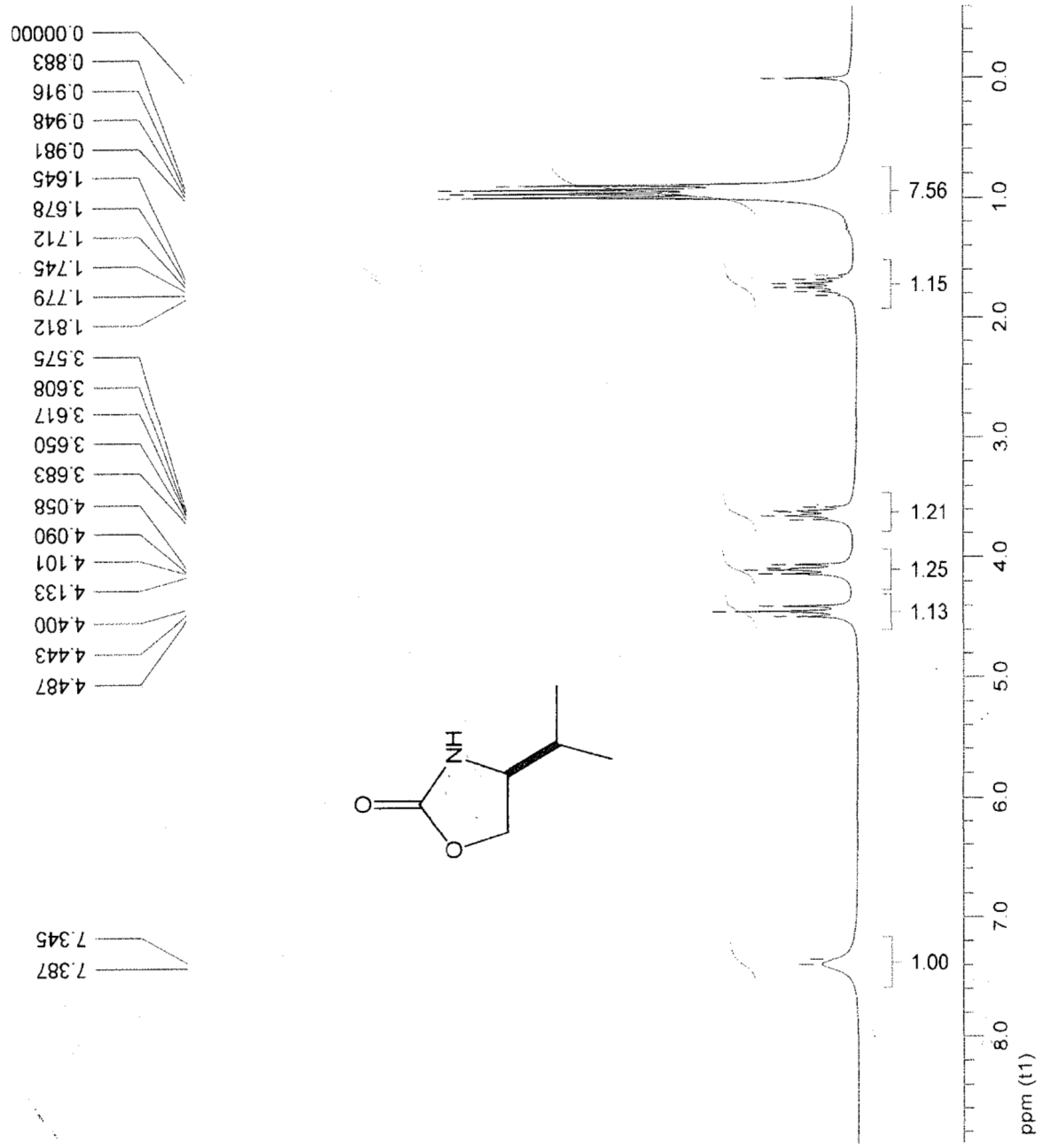

Figure S5. ^{13}C-NMR spectrum (75.4 MHz, $CDCl_3$) of Evans' oxazolidinone **2**.

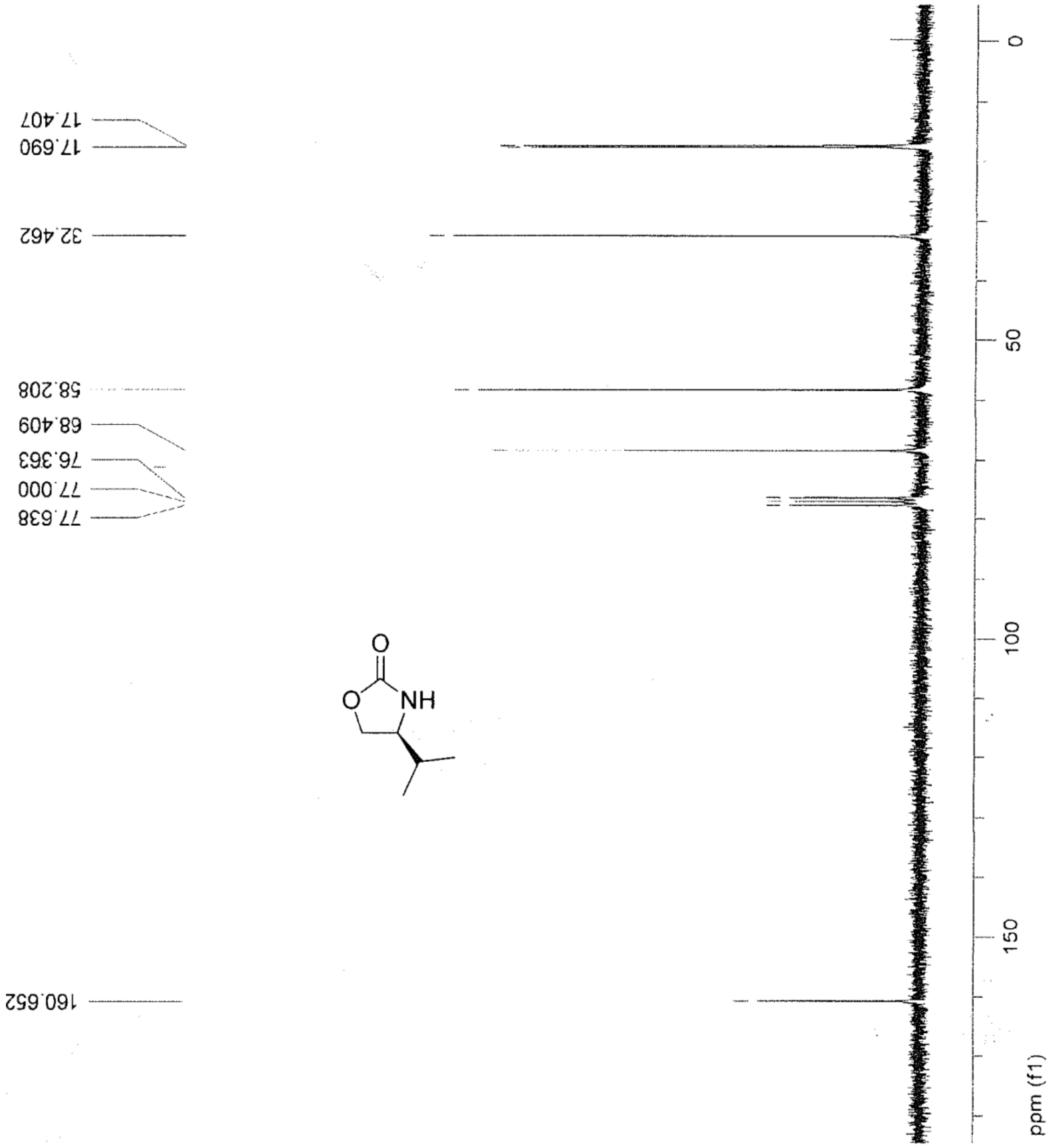

Figure S6. IV (KBr) spectrum of Evans' oxazolidinone **2**.

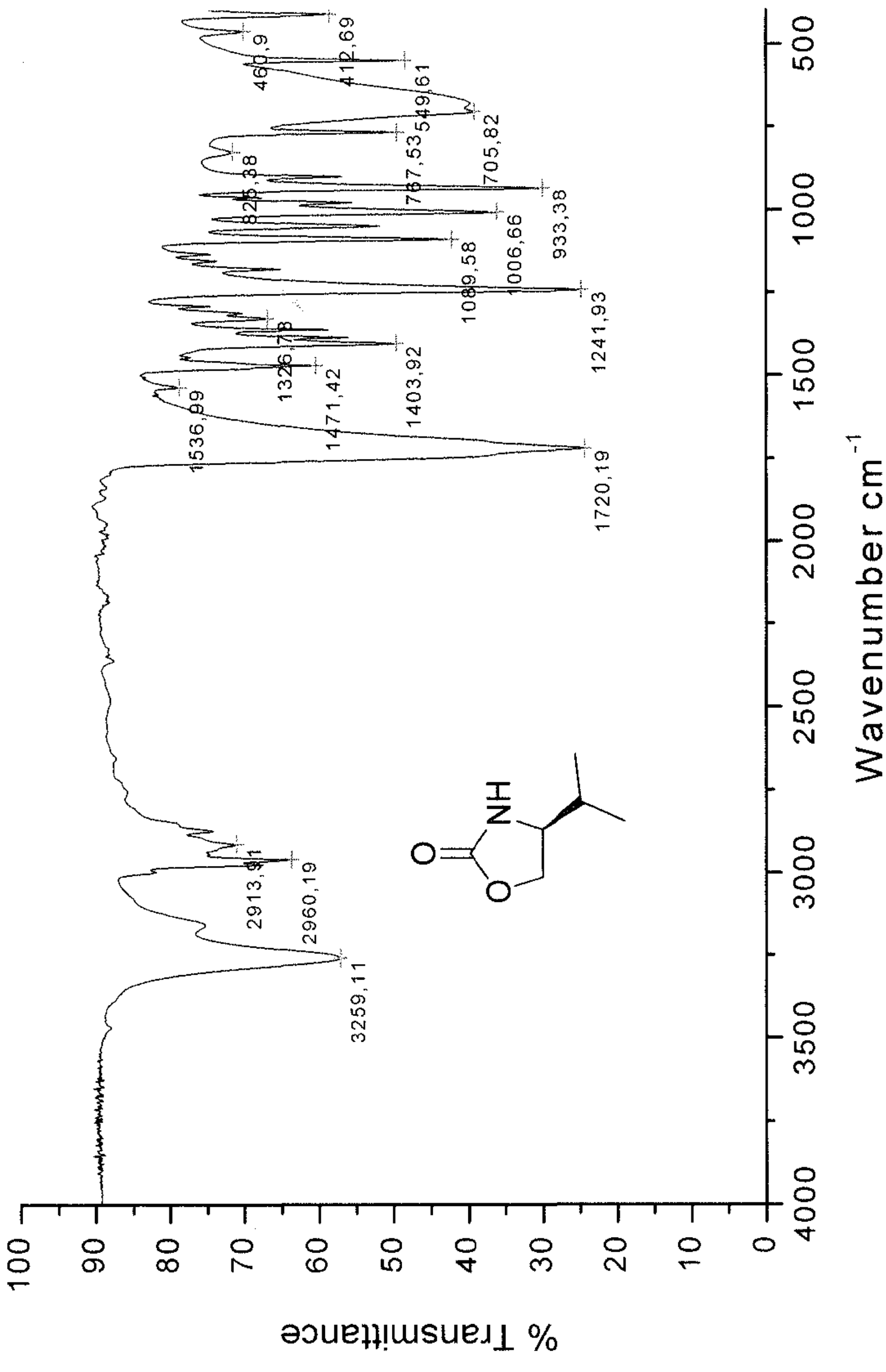

Figure S7. ^{1}H-NMR spectrum (300 MHz, $CDCl_3$) of MBH adduct **6**.

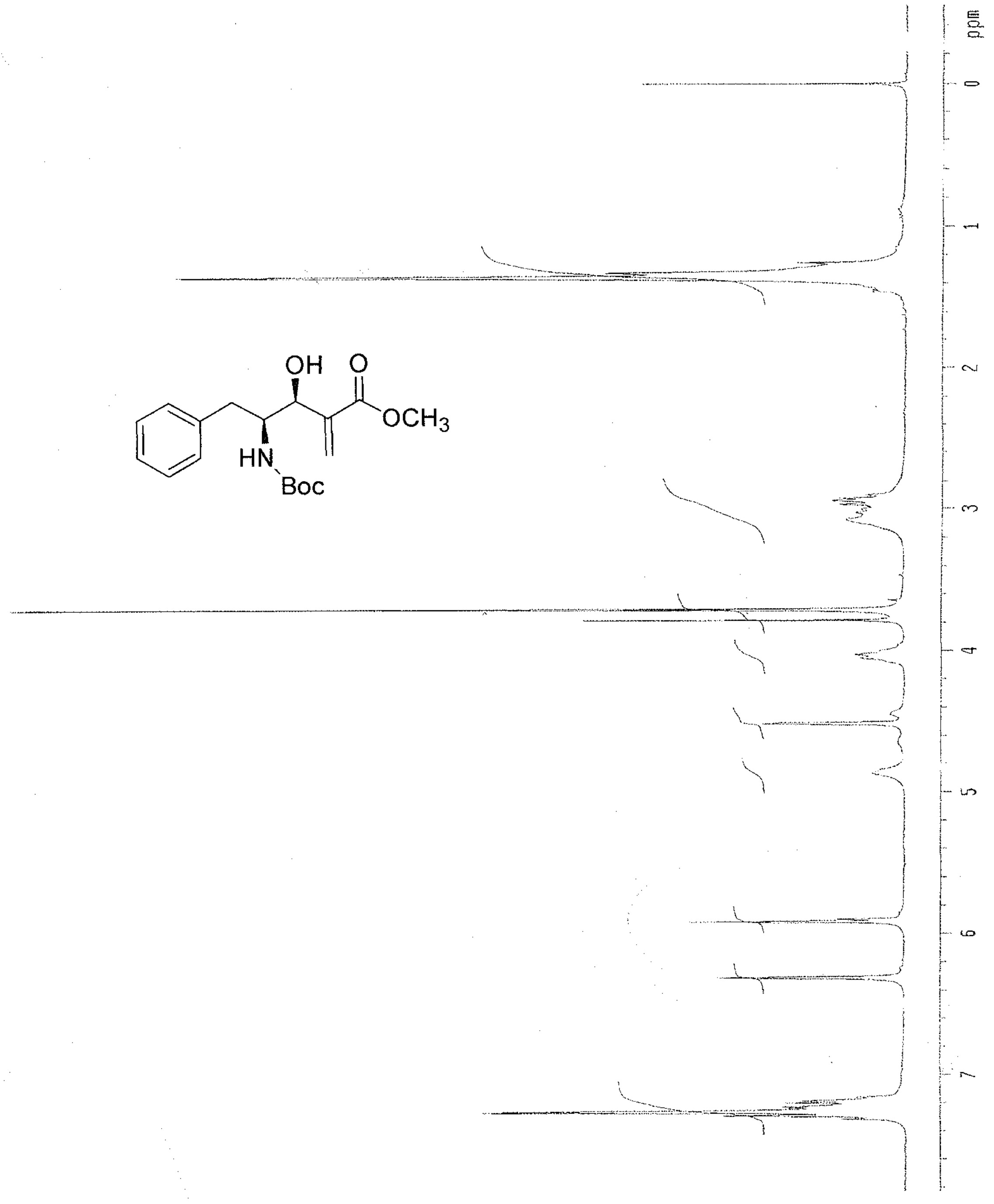

Figure S8. ^{13}C-NMR spectrum (75.4 MHz, $CDCl_3$) of MBH adduct **6**.

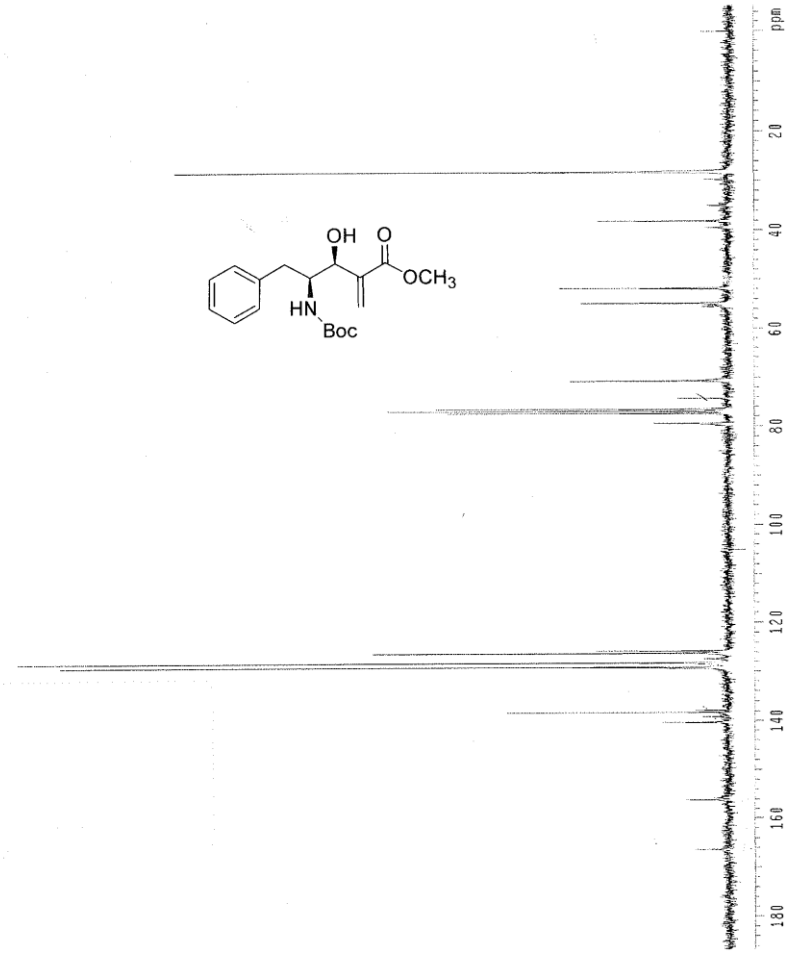

Figure S9. IV (film) spectrum of MBH adduct **6**.

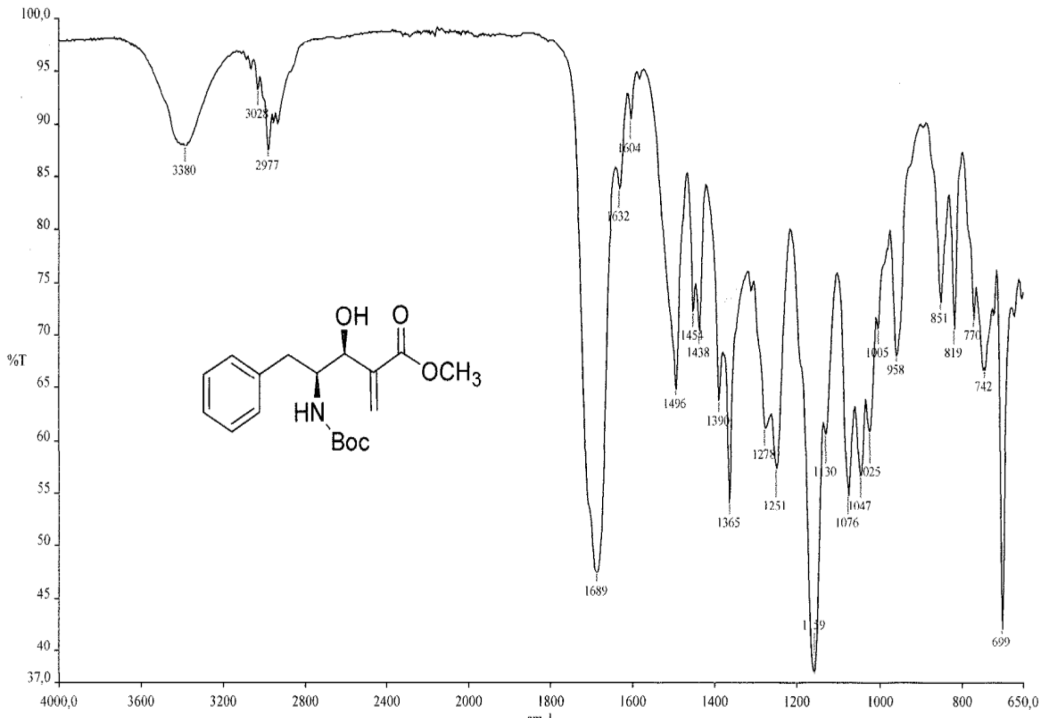

Figure S10. HRMS [ESI$^+$] *m/z* calcd for $C_{18}H_{25}NO_5$ [M + 1]$^+$: 336.1733, found: 336.1793.

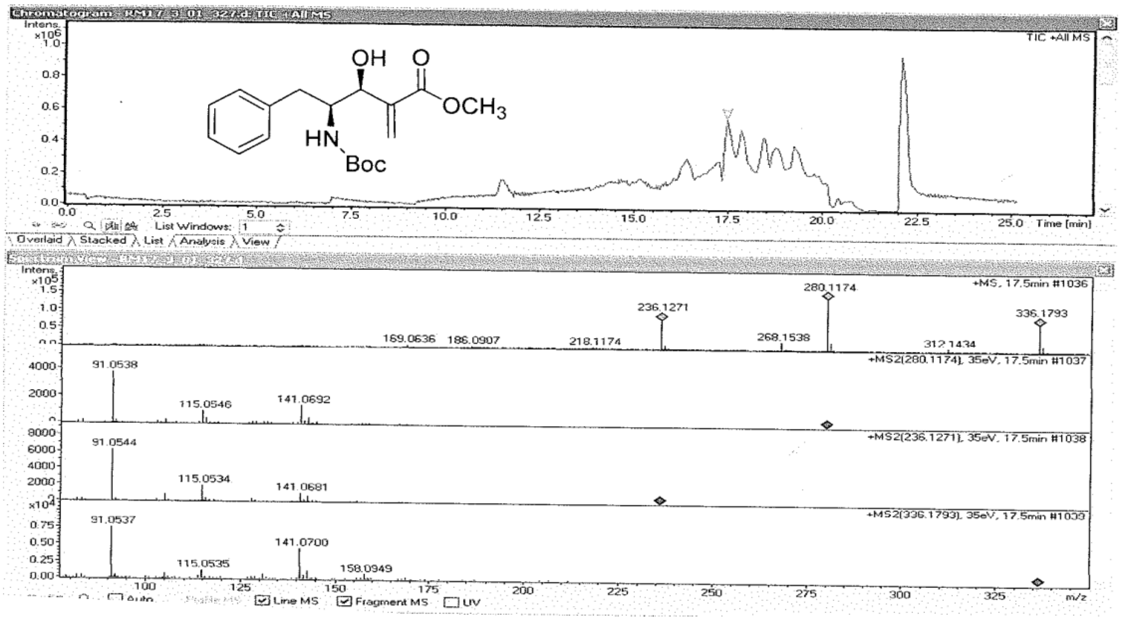

Figure S11. ^{1}H-NMR spectrum (400 MHz, $CDCl_3$) of acetonide **14**.

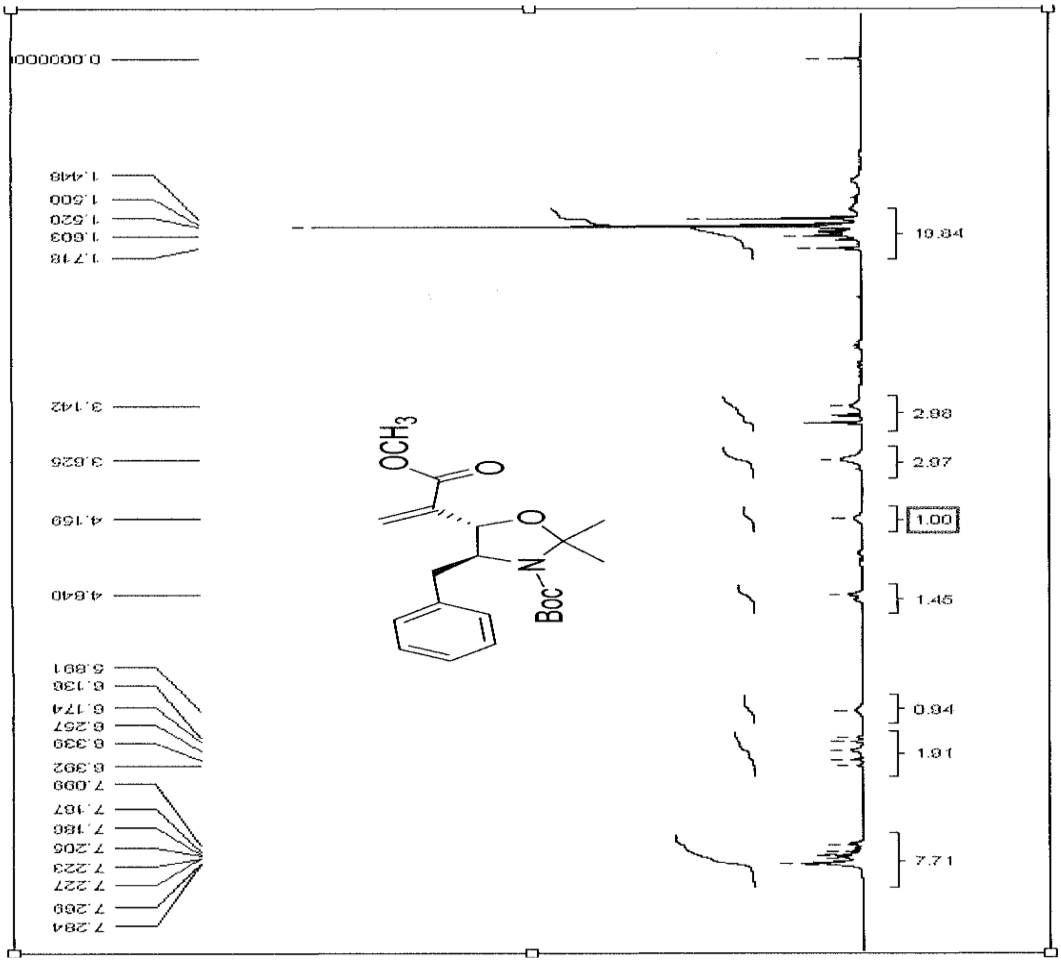

Figure S12. ^{13}C-NMR spectrum (100 MHz, $CDCl_3$) of MBH adduct **14**.

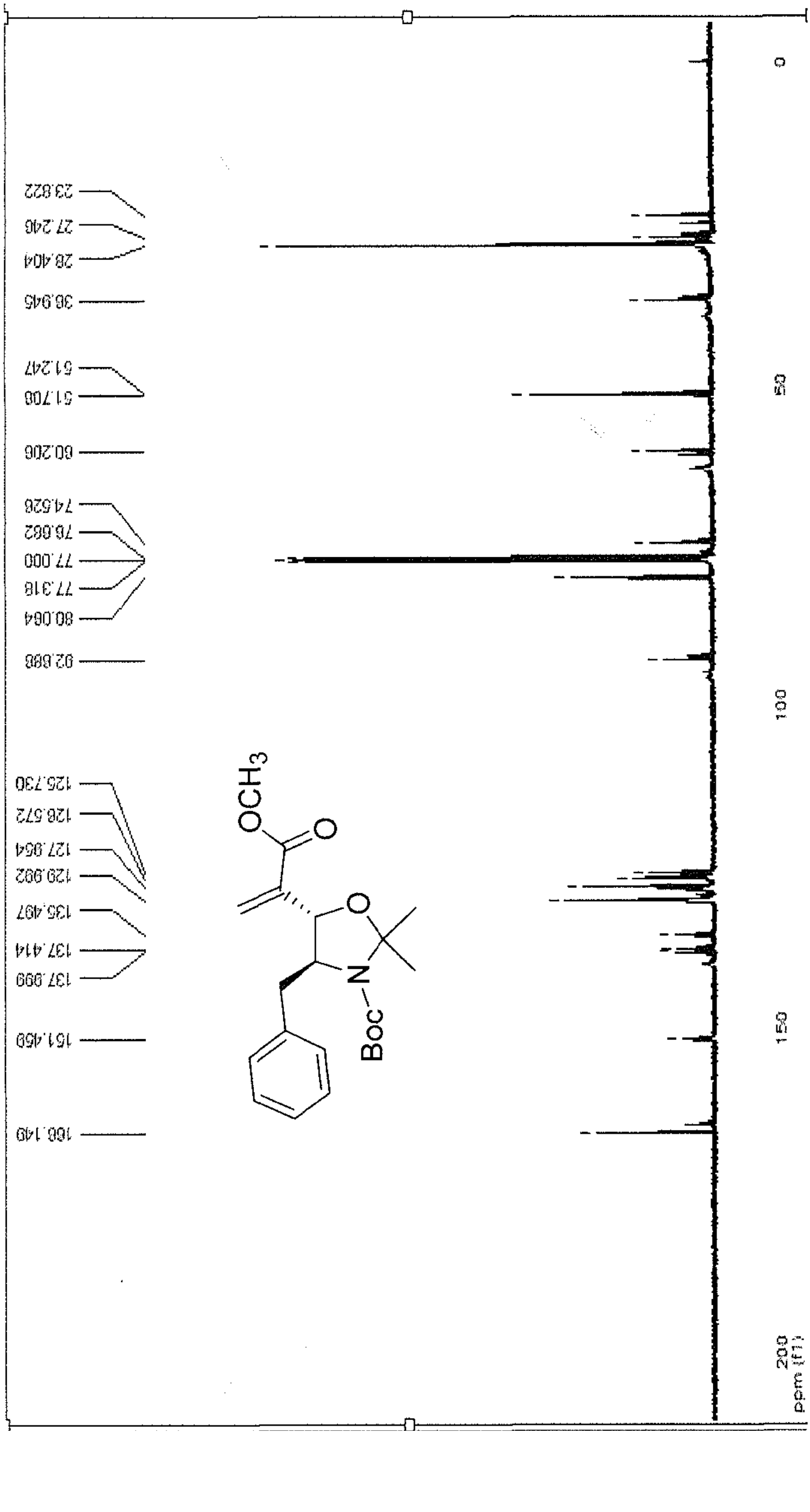

Figure S13. IV (film) spectrum of acetonide **14**.

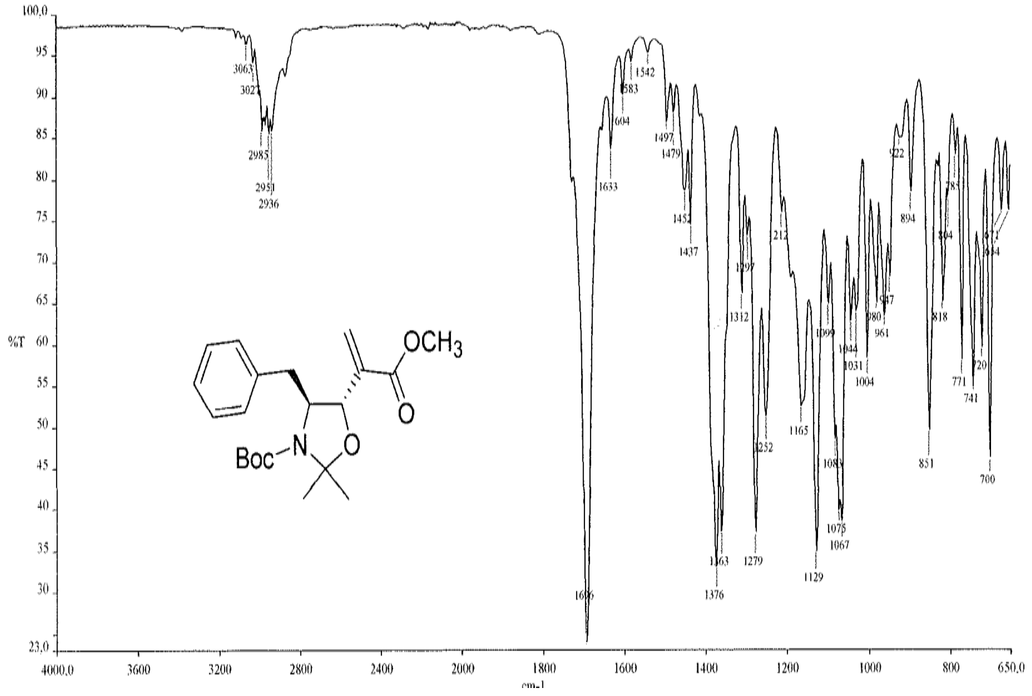

Figure S14. HRMS [ESI$^+$] *m/z* calcd for $C_{21}H_{29}NO_5$ [M + 1]$^+$: 376.2046, found: 376.2120.

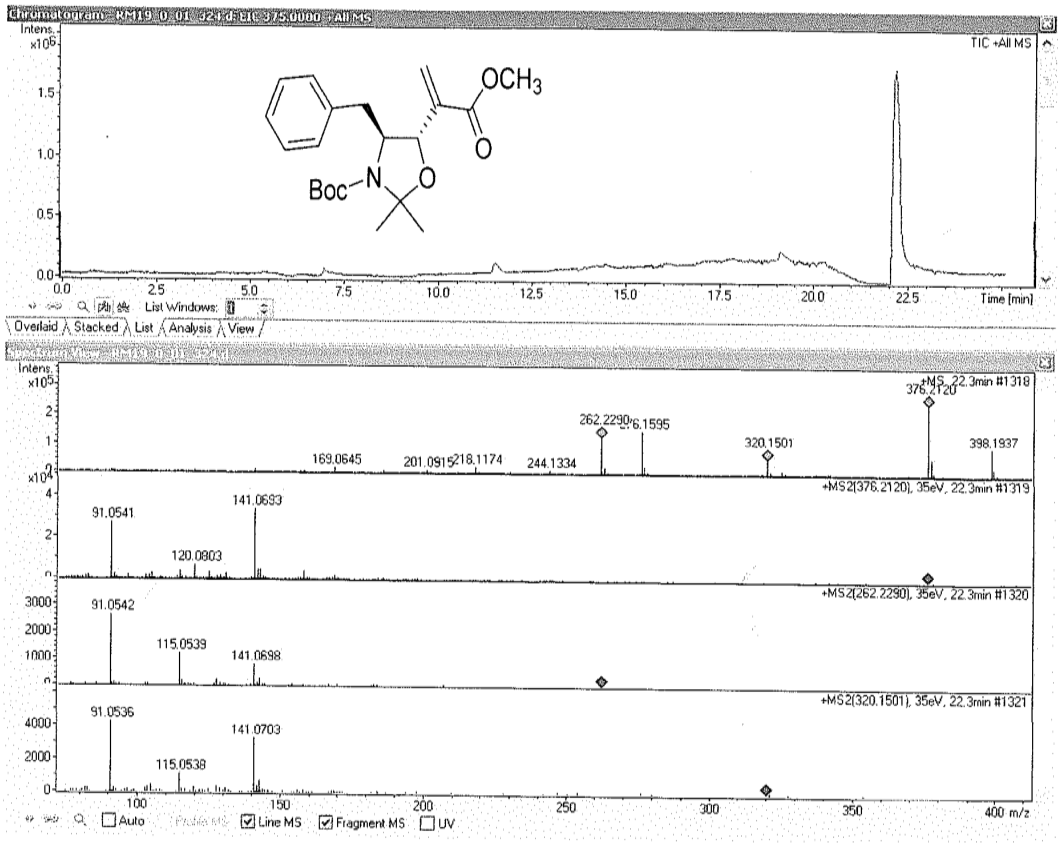

Figure S15. ^{1}H-NMR spectrum (400 MHz, $CDCl_3$) of oxazolidinone derivative **5**.

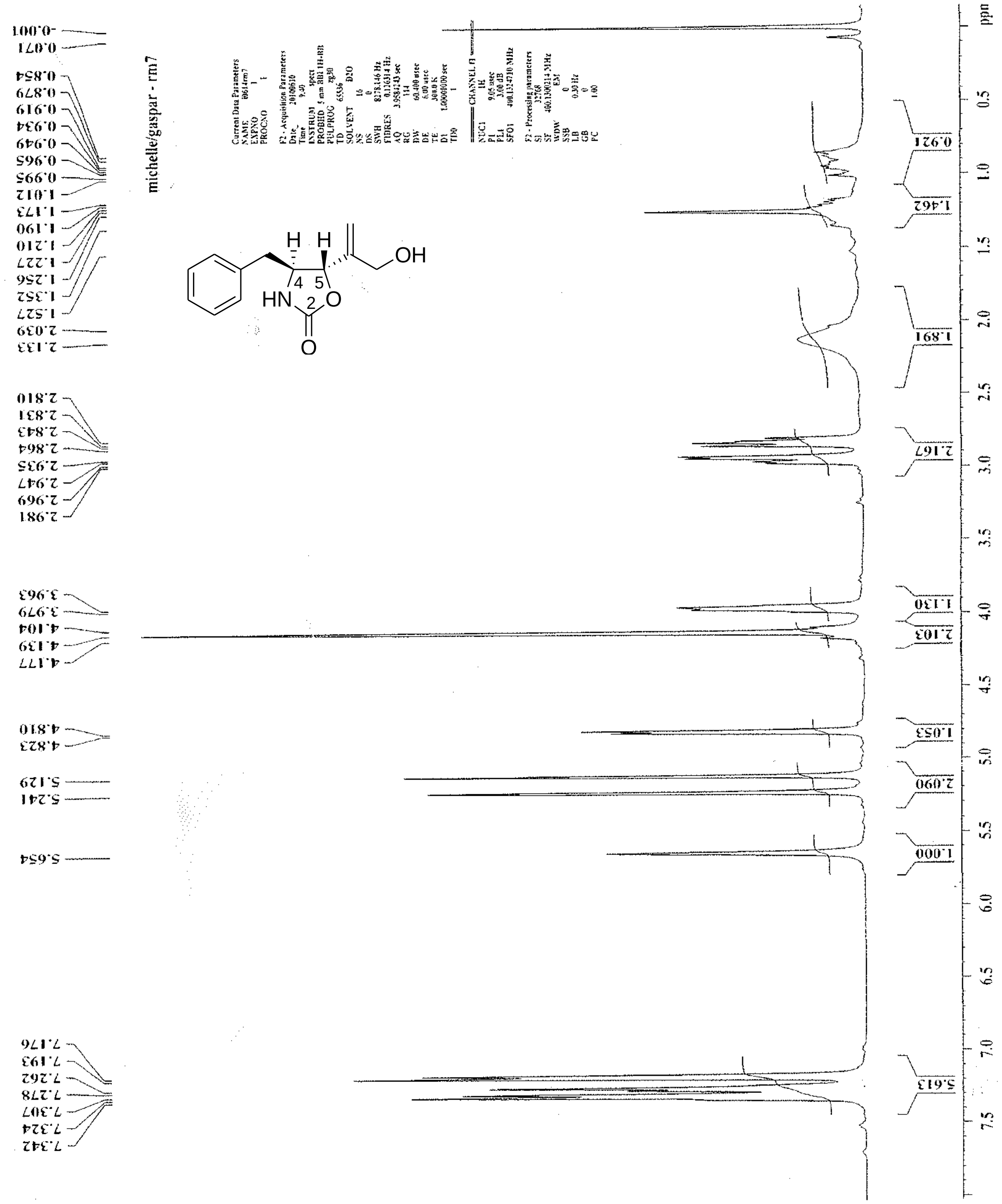

Figure S16. ^{13}C-NMR spectrum (100 MHz, $CDCl_3$) of oxazolidinone derivative **5**.

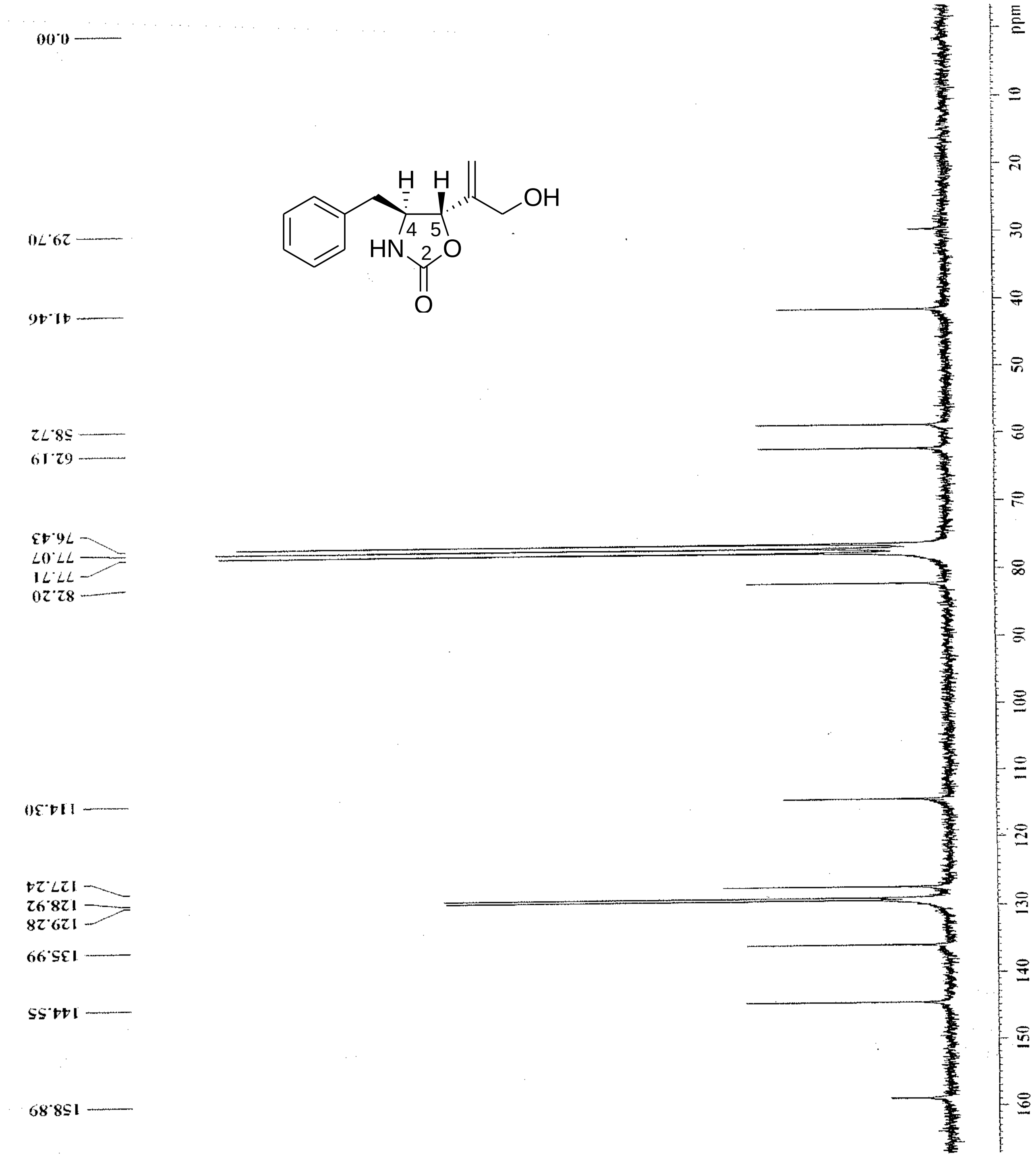

Figure S17. IV (film) spectrum of oxazolidinone derivative **5**.

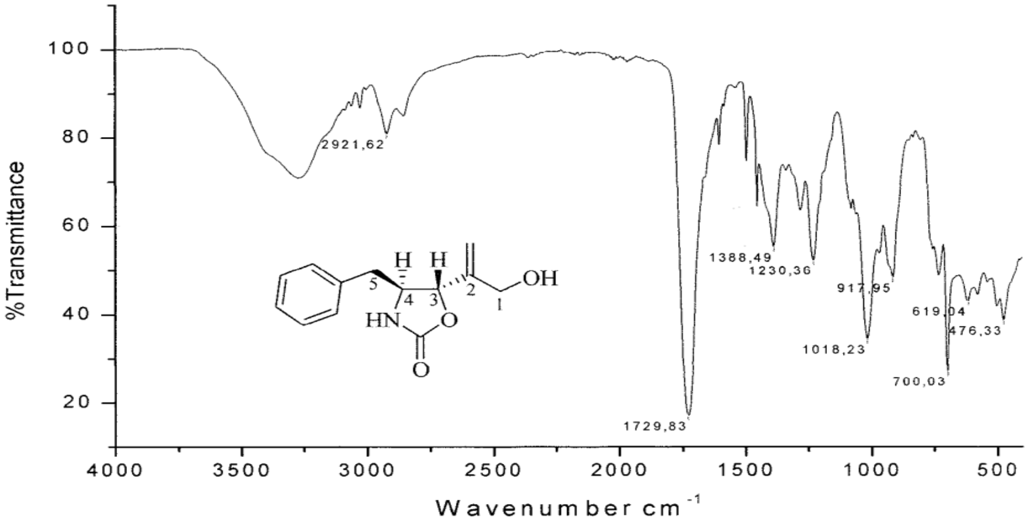

Figure S18. HRMS [ESI$^+$] *m/z* calcd for $C_{13}H_{15}NO_3$ [M + 1]$^+$: 234.1052, found: 234.1069.

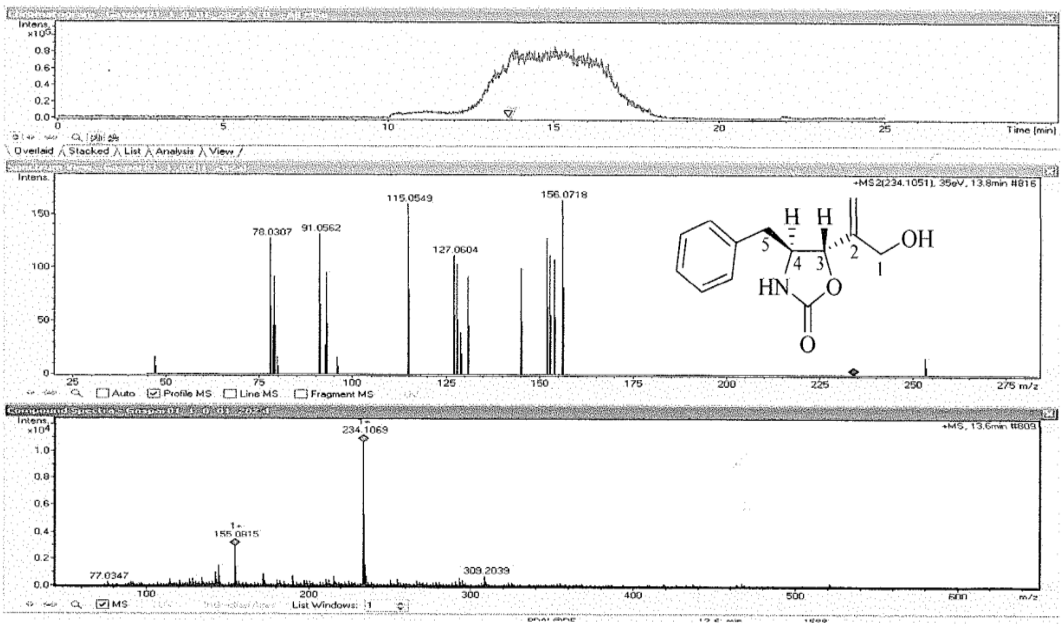

Figure S19. Chromatogram of MBH adduct **6**.

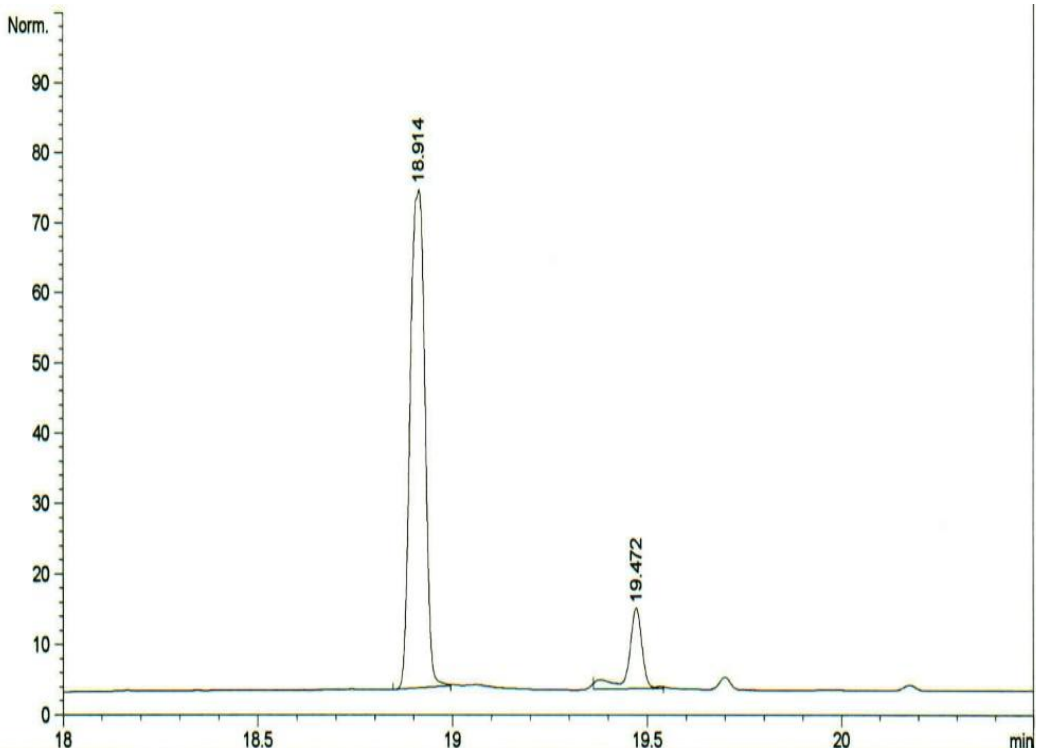

Conditions: (Column chiral HP (20% permethylated β-cyclodextrin): analytical condition: 100 ℃, 1 min; 100–150 ℃, 10 ℃/min, 150–250 ℃, 10 ℃/min.

Figure S20. Chromatogram of the oxazolidinone derivative **5**.

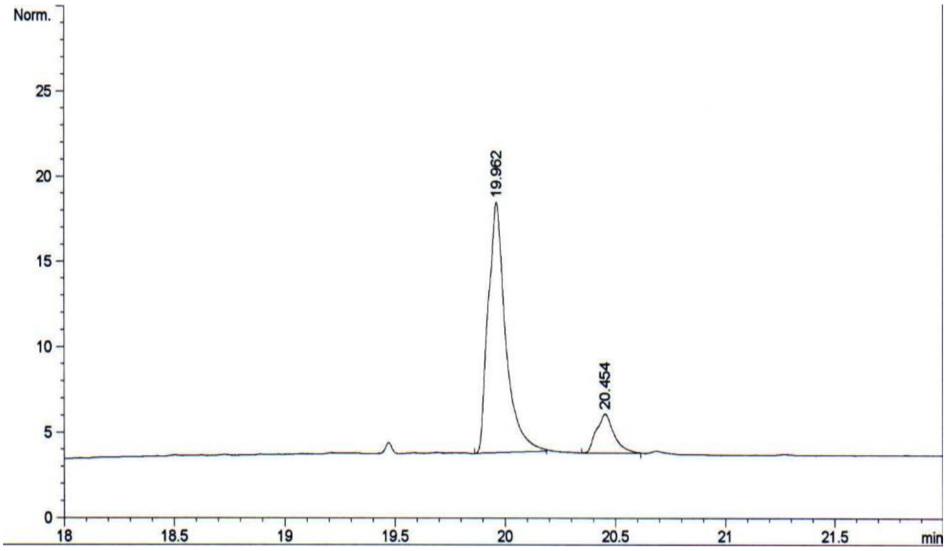

Conditions: (Column chiral HP (20% permethylated β-cyclodextrin): analytical condition: 100 ℃, 1 min; 100–150 ℃, 10 ℃/min, 150–250 ℃, 10 ℃/min.
